# Celiac disease and gluten-free diet: past, present, and future 

**Published:** 2020

**Authors:** Peter Makovicky, Pavol Makovicky, Fabian Caja, Kvetoslava Rimarova, Gabriel Samasca, Luca Vannucci

**Affiliations:** 1 *Czech Centre for Phenogenomics, Division BIOCEV, Prumyslova 595, 252 50 Vestec. Czech Republic *; 2 *Department of Biology, Faculty of Education, Selye Janos University, Bratislavska 3322, Komarno. Slovak Republic *; 3 *Laboratory of Immunotherapy, Institute of Microbiology, AS CR, v.v.i., Videnska 1083, 142 20 Prague 4. Czech Republic *; 4 *Department of Cell Biology, Faculty of Science, Charles University in Prague, Albertov 6, 128 00 Prague 2. Czech Republic *; 5 *Department of Public Health and Hygiene, Faculty of Medicine, Pavol Jozef Safarik University in Kosice, Trieda SNP 1, 040 11 Kosice. Slovak Republic *; 6 *Clinical Emergency Pediatric Hospital, Clinic of Pediatrics II, "Iuliu Hațieganu", Iuliu Hatieganu University of Medicine and Pharmacy, Crisan Street, 3-5 No 400177, Cluj-Napoca. Romania*

**Keywords:** Gluten-free diet, Celiac disease, Cereals, Small bowel, Nutrition

## Abstract

A gluten-free diet is a special type of diet intended for people with celiac disease. The objective of this article is to report the past, present production, supply of gluten-free products as well as their future position in our market. In the past, there were only limited gluten-free products available and the awareness of the diet was limited to paediatric community. There were only few raw gluten-free materials and almost all the production was created in households. Later with the introduction of targeted screening into the practice, celiac diagnosis has improved, with an increase in newly diagnosed patients who have remained life-long dependents on a gluten-free diet. This was associated with an increased production of gluten-free products, referred to as weight loss diet, with their positive effects on health. Subsequently, the gluten-free diet has also been voluntarily adopted by both people with other diseases and healthy individuals. In the future, the gluten-free diet consumption is expected to increase, due to its increased popularity in populations. In this regard, gluten-free diets have been misinterpreted as a "miracle drug" that is effective on a variety of problems. The medical community will be confronting the future problems of people who are dependent on a gluten-free diet as well as the complications arising from the consumers of a gluten-free diet for no medical reasons. Compliance to the principles of a gluten-free diet should be maintained and should not be recommended to healthy individuals or those without relevant reasons.

## Introduction

 Celiac disease is a disease that affects people of all ages and is characterised by gluten intolerance. It is classified as autoimmune enteritis with positivity of transglutaminase 2 autoantibodies and the destruction of small intestine mucosa, accompanied by a wide spectrum of clinical symptoms. Extra-intestinal symptomatology is not considered unique to celiac disease in clinical practice, with the disease being often diagnosed in older patients (1). The association between gluten intake and clinical symptomatology has also been observed even in atypical forms of celiac disease (2). The therapy for patients with celiac disease is based on a strict gluten-free diet, and no significant changes to this have been applied in recent years (3). Gluten must to be completely excluded from the diet of hypersensitive patients. Gluten-free diets are now a hot topic among the professional community. Gluten is a protein complex that is found as a natural component of grain seeds and is thus very frequent in various foods, such as flour, as well as other products. A gluten-free regime also requires specific preparation; on the surface, it is a simple elimination of gluten in the patient’s meals, but in detail it should not be only limited to gluten restriction, as gluten-free diet is associated with some risks. In the 1970s-1980s, celiac disease was considered as a very rare children´s disease, and gluten restriction was relatively unknown by medical professionals and the general public. Later, research showed that this disease can also be common in older age groups. Atypical forms of celiac disease were also starting to be studied by other clinical sciences (4). Improvements in the diagnostics and screening increased the incidence of disease, which pushed food producers to introduce gluten-free products onto the market. This led to the widespread distribution of gluten-free products, boosted awareness and inevitably, a mass interest in them (5). Gluten-free diets have significantly helped celiac patients to restore the morphology of their small intestine (6). However, many healthy individuals also adopted gluten-free diets, which yielded new problems. Medical studies have disputed the association between gluten and other pathologies, though relevant proof is still missing. The aim of this article is to review and discuss the past, present, and future significance of the gluten-free diet, and its associated complications. 

## Methods

Increased numbers of people diagnosed with celiac disease has promoted gluten-free market production and raised awareness of this diet in the public. A gluten free diet is currently being implemented in many people’s lifestyles both healthy and the sick. This paper intends to review the way gluten-free diet has changed over the years. Here complications in a gluten-free diet are presented. This is shown from scientific references and our experiences. 

**Gluten-free diet in the past**

Samuel Gee was one of the first authors to describe the gluten-free diet (7). Its principles were described by a Dutch physician Willem Karel Dicke who observed improved prognosis in patients who avoided the consumption of gluten (8). In the Europe, paediatricians were first in contact with celiac patients. However, the knowledge about the celiac disease and gluten-free diet was very poor, including among professionals. A praiseworthy effort was made to understand this disease, leading to the development of new research programs, but it proved to be very problematic to provide gluten-free diet advice to diagnosed children and their parents. This was because a market of gluten-free products was non-existent, and no relevant information was available to those producing food. The most required product was gluten-free bread or at least, gluten-free flour where professionals suggested making gluten-free bread by mixing potato and corn flour. Another alternative was to use soy flour, which would also suffice for preparing cakes and desserts. However, these types of flour were very rare, which t the production of gluten-free food using new approaches (9). We would like to highlight that despite the fear of a new diet, the mothers of affected children made a valiant effort initially before many specialised organisations were established, initially by establishing contacts between parents, and later by non-official organisations. The request for gluten-free diet was the main accelerator that led to establishment of private companies and producers present in the gluten-free industry. 

**Gluten-free diet in the present day**

According to a European law, there are two categories of food defined by composition and labelling. The first category describes specially formulated foodstuff for people with gluten intolerance, and the second includes all food for common nutrition and healthy individuals. Products in the first category are labelled as “Gluten-free” with a maximal gluten dose of 20 mg/kg, or “Very low gluten”, which includes products with a maximal dose of 100 mg/kg. This directive is notorious among producers and sellers of gluten-free products and is based on the observation that low doses of gluten are not harmful to celiac patients. According to Collin *et al.* (10), 30 mg of gluten per day is safe for celiac patients. Catassi *et al.* (11) performed tests on 49 patients (37 women, 12 men) with bioptically confirmed celiac disease, where all participants were placed on a diet with a maximum gluten intake of less than 5 mg/day for two years. They were then divided into three groups, each taking pills with a different dosage of gluten, 0 mg/day, 10mg/day, and 50 mg/day, for a period of 90 days. One individual, taking pills with 10 mg of gluten, relapsed, and no significant differences were found in the intraepithelial lymphocyte distribution between these groups. The authors concluded that celiac patients should not consume more than 50 mg of gluten per day, and problems arose with this European directive when producers did not follow the maximal doses of gluten in their products. Further difficulties appeared in some individuals who were extremely sensitive to even lower doses of gluten (12), and others who seem to tolerate gluten only within very specific ranges of concentration. Clinical practice has shown that some patients can tolerate low doses of gluten in their food, while some do not (13, 14). On the other hand, the results of Lahdeaho *et al.* (15) documented that low amounts of gluten can also cause significant mucosal deterioration in the majority of the patients. Today, there are many producers in the gluten-free product market and their goods are generally separated from other items in supermarkets. Alternatively, wide ranges can be found in shops dedicated to healthy food and organised public markets. Buffet kitchens, restaurants, hotels, school canteens, and even airlines have begun to offer these meals and allergen information is routinely listed in menus. Gluten-free produce is also often seen with price reduction offers, and on various television advertisements, bulletins, posters, and billboards. The Internet is a useful tool that allows the spread of information about gluten-free products, and how to prepare this food. These sources of information have helped celiac patients maintain a better lifestyle, while also creasing new problems. We believe that gluten-free diets should be derived from natural products, rather than modified produce. According to the findings that low doses of gluten in a celiac patient’s diet can also be harmful, we suggested to update the European legislative (16). Although we were not successful in this effort, we think that it is important to inform people about gluten-free products in the media. The diet’s increased demand has also forced health insurance companies to reimburse consumers of some products, even when they would not have been typically. The new challenge is to persuade governments and health insurance companies for full reimbursement of basic treatments since some families may not be able to pay for therapies and products, leading to a decline in the patient´s health. Compensation from governments would have a positive impact on the socioeconomic status of celiac patients´ families, as well as protecting them from health complications that may appear (17). 

**Perspectives of gluten-free diet**

Famous people were asked questions in relation to the gluten-free diet, such as: “Do you think celiac disease can be cured independently of a gluten-free diet?”. Professor Marsh, known to general society as the creator of celiac disease classification based on histological observation, excluded this possibility (18). Professor Freeman, the head of Gastroenterology Clinics at University of British Columbia, did not agree with this statement, explaining that the gluten-free diet is the best solution at the moment, hoping his opinion is correct (19). According to a respected specialist from the B. Rappaport Institute, Professor Lerner, the importance of the gluten-free diet will only increase in the future. Even if new immunotherapies based on vaccination will cease autoimmune reactions in small intestine mucosa, it will take a very long time to confirm the real effectivity of this therapy, including all side effects. A gluten-free diet will remain the most effective and faithful therapy of celiac disease (20). Dr. Rostami-Nejad, the head of Celiac Disease Department of Gastroenterology and Liver Diseases Research Institute of Shahid Beheshti University of Medical Sciences in Iran, is more optimistic and he believes that vaccination will replace current gluten-free diets (21). Professor Arato, a valued professor of Department of Pediatrics at Semmilweis University in Budapest since 1977, shares this opinion. He believes that modulation of innate immunity can influence the lymphocytes that primarily recognise gliadin and transglutaminase, and that gluten-free diets will be improved by alternative therapy (22). Also, Assistant Professor of Paediatrics Dr. Hakim Rahmoune from University of Setif-1 & University Hospital of Setif in Algeria is very optimistic about alternative celiac disease treatments. He strongly trust in the power of the gastrointestinal microbiota in celiac disease treatment (23). These opinions are just preliminary to the current situation in the field, and only future experiments and studies can prove their validity. Currently, celiac disease therapy is very problematic without gluten-free diet. This fact is well-known to all professionals in the field while many studies are now running. 

**Gluten-free diet for healthy individuals**

As we discussed in the introduction, a surge of newly-diagnosed celiac patients led to increased awareness of gluten-free diets among the public, and numerous information sources helped this revolution. First of all, celiac patients are dependent on this diet for the rest of their lives, and celebrities with celiac disease have become the target for all media platforms. Many popular books about this topic have gained such a wide interest that they were translated into many different languages. For example, Perlmutter (24) mentions many health problems associated with celiac disease, including complications and pathology that are caused by gluten intake. Another brings suggestions on how to lose weight with a healthy life-style (25). A book about the life of Novak Djokovic, mentions that a gluten-free diet helped him achieve his triumphs in his sporting career (26). These are examples of publications that have helped to popularise gluten-free diets not only among patients but also in the general population. Specifically, 1 in 10 families in the USA have one person diagnosed with gluten intolerance, but one third of the country’s population have decided to adopt a gluten-free diet (27). According to one US study, wealthier families are more likely to make this decision, and one third of them are not able to give any reasons for this preference. However, it is believed to be only one of many healthy diets that helps them sustain their body weight (28). More healthy people are now following a gluten-free regime, but it was primarily proposed for celiac patients. The links, or potential health problems between gluten-free diet and people without celiac disease are not well known (29). Indeed, the present general trend is to follow a gluten-free diet. The reasons for this are varied and is based on the above-mentioned sources of information. Professionals need to inform their patients to follow all principles of the diet, stating the risks and complications if the rules are not upheld. 

The production of gluten-free food represents a vast part of the food industry. This suggests that the popularity of gluten-free diets will only increase, and will be more significant than the increase in the number of diagnosed patients, leading to widespread gluten-free diet consumers in our population too. One study focused on 155 healthy individuals on gluten-free diets and compared them to celiac patients (30). The results showed that healthy volunteers were mainly women with slim waists, higher blood HDL cholesterol, and significant weight (1.33 kg/year). Another study dealing with gluten-free diet in French adults without celiac disease revealed that women, older persons, non-smokers, and people with lower educational level followed this diet (31). We were also asked for our opinion on how important a gluten-free diet is for the healthy population. According to experimental studies, a gluten-free diet combined with a classic diet is far healthier than a strict gluten-free diet (32). In our experiments, we compared the length of the villi and depth of the glands in the duodenum of conventional Wistar rats, which were exposed to gluten-free and gluten diets, and then a combination of both. Although this was a preliminary experiment, the results indicated that a combined diet increased the length of the villi in comparison to the other experimental groups. We can conclude that gluten-free diet is not suitable for healthy individuals. 

**Complications accompanying gluten-free diet**

Long-term gluten restriction can induce many health risks, including nutritional deficiency (33), cardiovascular problems (34), as well as an accumulation of heavy metals in the organism (35). Thus, all celiac patients have to be informed about these health risks, which are well known among professionals (36). These are also valid for healthy people following a gluten-free diet, and must be made aware too. Gluten-free diets are rich in lipids, sugars, and salt, providing a higher energy intake in comparison with a normal diet. This can be considered harmful, and is not recommended for healthy consumers by professionals (37, 38). Gluten-free diets can contribute to even more health problems, including obesity, dyslipoproteinemia, insulin resistance, metabolic syndrome, or atherosclerosis (39). For this reason, a new solution is required for quality of life of celiac disease patients, but not for celiac disease treatment. Health education on gluten-free diet in the society seems to be the solution (40). Gluten is extracted from surplus products of the starch industry whose structure and nutritional properties are very valued in human and animal diets. Some studies have shown that gluten increases intestinal permeability, oxidative stress, has anti-inflammatory properties, and decreases the differentiation of intestinal cells (41). This information is not properly distributed by non-professionals, and claims that gluten is extremely harmful. On the other hand, observations also highlight the importance of gluten in our diet, and should not be missing if people are to remain healthy. There are significant differences in gluten harmfulness between healthy and intolerant people. While in sensitive individuals it induces a cascade of reactions leading to an inflammatory condition in the small intestine, with atrophy of villi and other health complications, healthy individuals can develop dyspeptic complications, which are not associated with gluten intolerance. These people need to be investigated and diagnosed for the cause of the problem. If health problems of these patients are not caused by gluten itself, gluten-free diet is not the therapeutic solution. 

**Gluten-free diet in therapy of other diseases**

An attentive reader may find information in the literature that certain genetic factors associated with celiac disease are also common in other diseases. Thus, a gluten-free diet may be helpful in these cases (42). Often, the literature mentions the positive impact of this diet on health. For example, it can help not only in metabolic syndromes (43), but also in other autoimmune disorders, including lymphocytic duodenitis, non-specific duodenitis (44), refractory inflammatory bowel disease (45), while also improving insulin secretion in type 1 diabetes mellitus (46). An association between gluten and psychiatric disorders has also been demonstrated (47), showing the protective effects of a gluten-free diet in patients with schizophrenia, and causing an improved outcome and tolerance (48, 49). To introduce the gluten-free diet in the therapy of other diseases, it is important to follow the same criteria as for celiac disease. Gluten must be involved in the pathogenesis of the disease and its elimination must bring some therapeutic effects. It has not been definitively proven that gluten is involved in the pathogenesis of other diseases yet, and will take more time to find these pathogenic associations. Also, further investigation is required to prove the importance of a gluten-free diet for their therapy, for example in autoimmune diseases (50). The growing interest in gluten-free diets from the general population brings new health problems with its consequences. Physicians will play an important role of explaining the associations between the disease and gluten to their patients, and when deciding for a gluten-free diet, to clarify how to follow the lifestyle connected with it. 

## Conclusion

This article summarised our differing opinions of gluten-free diets and the associated risks. It is a well-known diet between ordinary and professional societies. Gluten-free diets are a special form of diet for patients with celiac disease. Previously rare groups of products have now become available in a wide range in all major shops, and intensive advertisement has stimulated an increased production of them; we suppose its popularity will further rise in the future. Many specialists hypothesise that gluten-free diets can be helpful in other diseases too and despite the effort to advocate this diet to celiac patients only, we can expect it will be gradually more popular not only among patients with different diagnoses, but also in healthy individuals. The risks associated with gluten-free diet are still intensively discussed among specialists and have remained a major problem in clinical practice. This article intended to aid in illustrating the relevant specialist advice that patients will require to fully understand their syndrome.
